# Supplementary material for: Isolation and Establishment of a Highly Proliferative, Cancer Stem Cell-Like, and Naturally Immortalized Triple-Negative Breast Cancer Cell Line, KAIMRC2
Source: Cells. 2021 May 24;10(6):1303. doi: 10.3390/cells10061303 (PMC8225085; doi:10.3390/cells10061303)
Supplement: Supplementary file 1 [file cells-10-01303-s001.zip › cells-1180582 Supplementary Figures.pdf]

Pathways related to up-regulated genes

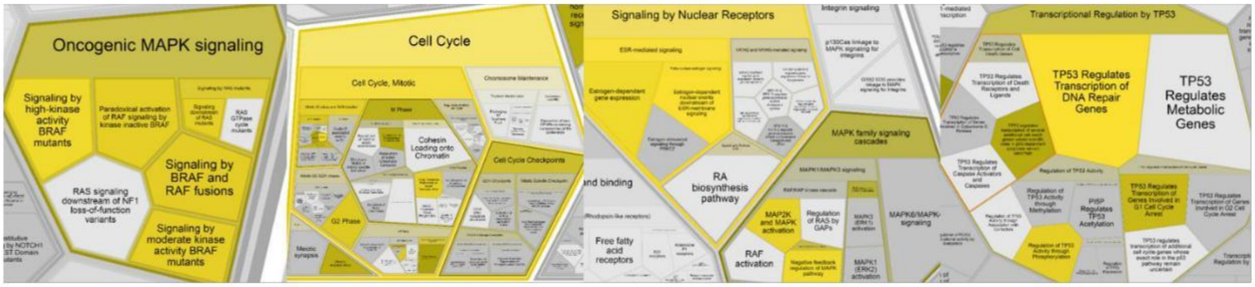

Pathways related to down-regulated genes

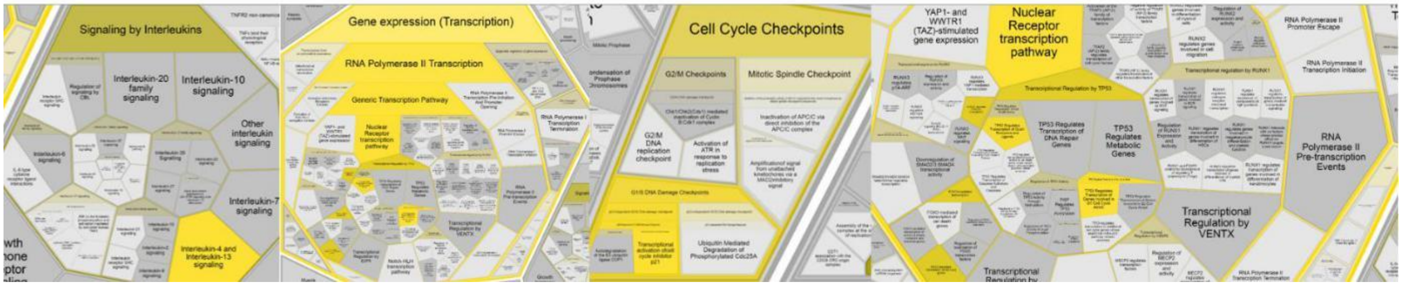

Supplementary Figure S1: A web-based pathway analysis software Reactome revealed the pathways related to down and up-regulation of key breast cancer genes in an interactive diagram.

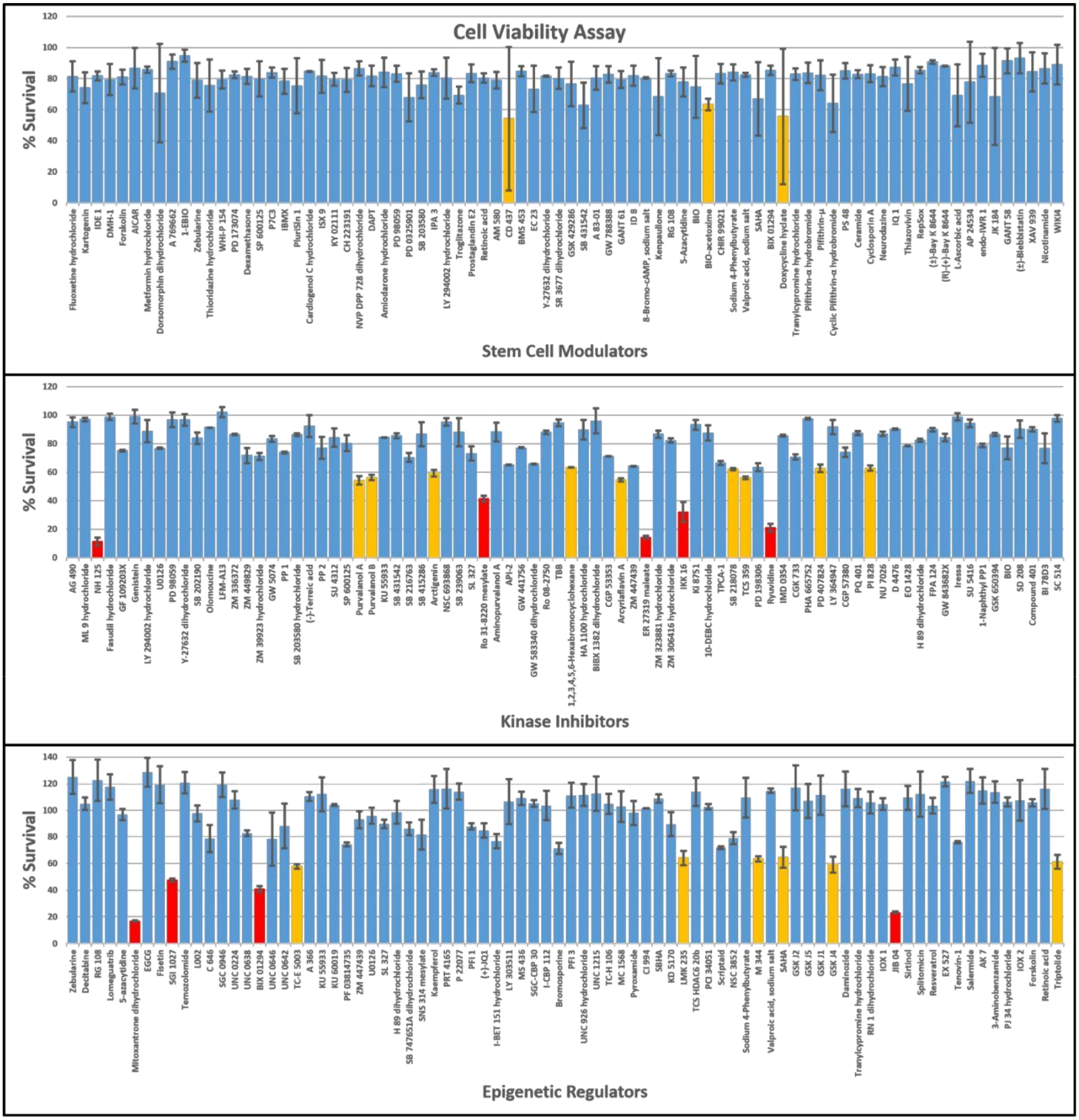

Supplementary Figure S2: This figure shows all the compounds used in this study for screening drug candidates for breast cancer KAIMRC2 cell line.

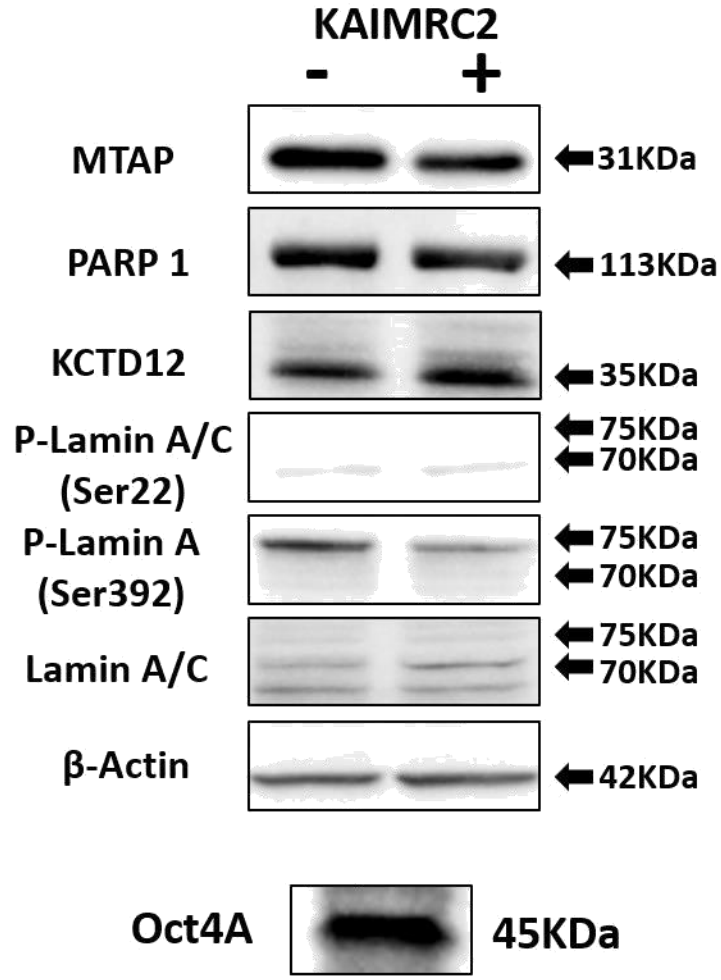

Supplementary Figure S3: Western blot analysis of a few of the proteins that showed a similar pattern of expression compared to the KAIMRC1 cell line.

Isolation of Breast Cancer Stem Cells

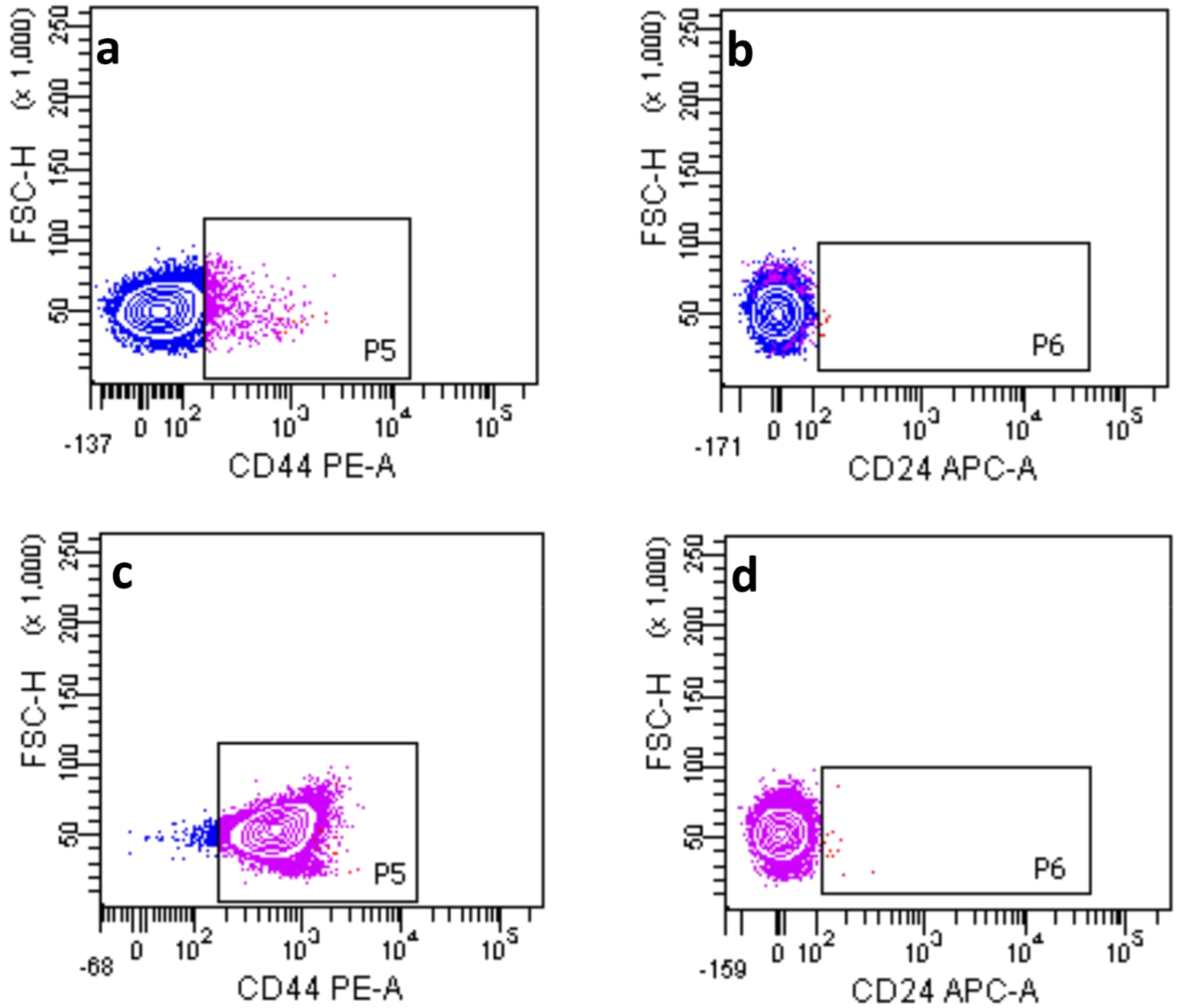

Supplementary Figure S4: Isolation of CD44+ Breast Cancer Stem Cell Population. (a, b) Unstained sample.

(c) CD44+ population of KAIMRC2 cells after MagCellect Isolation and (d) shows that the cell population is CD24−. ×20.000 cells were used for the flow analysis.
